# Restrictive Medical Legislation

**Published:** 1898-07

**Authors:** 


### EDITORIAL DEPARTMENT.

F. E. DANIEL, M. D., Editor.
S. E. HUDSON, M. D., Managing Editor.
A. J. SMITH. M. D., Galveston, Associate Editor.

Published Monthly at Austin, Texas, by Drs. Daniel and Hudson. Subscription price $1.00 a year in advance.

Eastern Representatives: Messrs. Monihan & Fairchild, St. Paul Building, 220 Broadway, New York City.

Official organ of the West Texas Medical Association, the Houston District Medical Association, the Austin District Medical Society, the Brazos Valley Medical Association, the Galveston County Medical Society, and several others.

### RESTRICTIVE MEDICAL LEGISLATION.

Under this caption Mr. B. O. Flower, of Boston, — once editor of *The Arena*—has, in *The Arena* for June '98, a screed which teems with evidences of prejudices against the "regular" medical profession,—false accusations, sophistry—bizarre ignorance of the subject, and special pleading for homeopathy and cognate frauds and shams. His argument, if it can be dignified by the term, embraces the stock objections of the homeopaths, and others who would "feel the halter draw" if an examination were required by the State, and was doubtless learned at the feet, or at the hands of that other, and malodorous "blossom"—his brother, I take it—who, from his eirie in Boston, annually swoops down on Texas, and incontinently does up the credulous and unsuspecting who are afflicted with cancer, consumption and other incurable diseases, taking—all that can be got out of them. It is the weakest thing I ever saw emanating from an alleged respectable source.

The mainstay and prop of Mr. Flower's tirade is the assertion that medicine is not a "science," but an art; that it is "not an exact science," and *therefore* the State should *put no restriction* on the practice! He stultifies himself in the next paragraph by going into rhapsodies over the alleged efficacy of—"Christian Science!"

Imprimis: Mr. Flower is reminded — if he ever knew it—that "science" is *applied knowledge.* If medical and sanitary knowledge is not "applied" in the prevention and cure of disease by the medical profession, then "science" has no meaning. Can Mr. Flower say that "Christian Science" is "an exact science," or that *knowledge* of *any kind* is applied in the alleged "treatment" of disease by such mummery? He reminds us that in Boston the "Christian Scientists" have "churches" in which "no preaching is allowed," but says that the devotees, the "Christian Science" apostles go there and "read the. Bible and pray." We can imagine the comfort and inspiration one of his sort would derive from a close perusal of the chapter telling of Lot's drunkenness and the debauchery of his own daughters, or of the exploits of Jehu, for instance. But will Mr. Flower tell us that the *prayers* cure?—that these Boston fanatics really induce God to weaken and relent? as these fellows pretend to believe that affliction is sent on man by the Almighty for his sins?

Is steam navigation "an exact science?" Yet no engineer or pilot will be permitted to take charge of a vessel without an examination and a license; nor will a locomotive engineer be entrusted with a train until he has given evidence of capacity and training which entitle him to confidence. Is military surgery "an exact science?" The government will not permit a doctor to deal with the life and health of its soldiers without a strict examination and a commission. (We assume that this fact, which seems to stick in Mr. Flower's throat, like Macbeth's "amen," is the real trouble with Mr. Flower; the "homos" can't come up to the standard, don't dare try, and hence there are no homeopathic "surgeons"—pardon the paradox—on the United States army or navy medical staff; and none of his brother's sort either, those peripatetic all around "doctors" or cure alls).

Mr. Flower's strongest "holt" though, seems to be "personal rights." He says that "every person should be permitted to select his own medical attendant," and every man the right to practice! By the same kind of reasoning all the children should have the "right" to pick berries in the woods, or to monkey with any kind of dangerous things ("not knowing they are loaded"), and not being able to distinguish and reject the poisonous berries any more than are the dear people able to detect the ignoramus and smooth fraud who, like the Boston "blossom," sounding his own praises in the *newspapers*, easily deceives the ignorant and credulous. Does Mr. Flower need to be told that personal rights are secondary to the rights of the people—the community, and that no man can be, or is, permitted to exercise his personal rights to the detriment or danger of others? The drunken rowdy would exercise *his* right to tank up, and run his horse through the streets, firing off his six-shooter right and left, were he not restricted by the law. He would have as much "right" to do so, as has an uneducated, untrained man or woman to undertake the treatment of disease or wounds; and it would be attended with no more danger to "the other fellow." The street boy has the "right" to throw stones, but not at my windows. Every man has a "right" to keep a powder magazine on his premises—if the law would let him.

This State guards the exercise of all dangerous privileges—dangerous to the public health—by throwing restrictions around them, except the most dangerous—the practice of medicine.

If Mr. Flower's ideas of personal rights were carried out, there could be no quarantine. Who would dare stop a sovereign citizen on his travels? Who dare open his trunks and smoke his good clothes with horrid sulphur?

The crowning absurdity of Mr. Flower's long paper—29 pages of the most unadulterated rot I ever toiled through—is, he cites the failure of "New York physicians" (despised "regulars," of course) to correctly diagnose the late Professor Proctor's case; (they called it yellow fever and had him insolated; he died,)—as a *reason* why every ignoramus—in the exercise of his "personal rights"—should be allowed without let or hindrance to practice medicine; to prescribe unknown drugs (unknown except by name) in any doses, for diseases of which they know nothing; to be entrusted with the knife and saw and trephine; the obstetric forceps and the craniotomy tools; to attempt operations they never saw done and know nothing of, except what they have read perhaps in some medical journal, for it is even a known truism and applies to homœopathy with peculiar force:

"Fools will rush in where angels dare not tread."

What greater danger to the health and welfare of a community than a daring "doctor," ambitious perhaps, or needy, but ignorant?

The State *unquestionably* has the right, in the exercise of its police powers;—nay, it is in duty bound—to put restrictions on the privilege of practicing medicine; such restrictions as will insure that that dangerous prerogative is confined to men who have been educated to the science of medicine, and who can and must be made to show to some State authority that they are competent to practice medicine. An ignorant doctor is as great a danger to a community as an epidemic of yellow fever—especially if he be popular; he's "loaded," but who knows it? And the State owes it to its children—the people—to protect them against this, as against all other subtle or open dangers.

Mr. Flower accuses the regular profession of seeking self-protection in their efforts to secure restrictive medical legislation. This is as contemptible as it is false and unjust. Why, if every homeopath in Texas were wiped out, or put to honest labor, and their practice was equally divided amongst the "regulars," it would amount to $3.70 per annum per capita;—we made the estimate once on a basis $2000 a year practice to every homeopath in Texas. The accusation is dismissed as too contemptible for further notice.

Mr. Flower says there are ignorant and incompetent men in the medical profession (regular). Precisely; and *because* of the lack of such laws as will require every candidate for the degree M. D. to show that he is as thoroughly educated as the best schools can educate him, and to show, also to the State where he seeks to practice, that he is educated, before he will be entrusted with a *license*. And until such laws are passed there will always be the danger of the "deadly doctor."

Mr Flower, you need, in this age of learning and advancement to be ashamed of some of the sentiments expressed in your *Arena* article, so clearly in the interest of quackery and humbuggery, and seemingly a reflex of and excuse for the unethical practices of the great Boston "cure all" of your name.

